# Effect of vitamin D supplementation in combination with weight loss diet on lipid profile and sirtuin 1 in obese subjects with vitamin D deficiency: a double blind randomized clinical trial

**DOI:** 10.15171/hpp.2019.36

**Published:** 2019-10-24

**Authors:** Soodabeh Aliashrafi, Seyed Rafie- Arefhosseini, Lida Lotfi-Dizaji, Mehrangiz Ebrahimi-Mameghani

**Affiliations:** ^1^Student Research Committee, Faculty of Nutrition and Food Sciences, Tabriz University of Medical Sciences, Tabriz, Iran; ^2^Nutritional Biochemistry, School of Nutrition and Food Sciences, Tabriz University of Medical Sciences, Tabriz, Iran; ^3^Social Determinant of Health Research Center, Faculty of Nutrition & Food Sciences, Tabriz University of Medical Sciences, Tabriz, Iran

**Keywords:** Lipid profile, Obesity, Sirtuin1, Vitamin D, Weight loss diet

## Abstract

**Background:** Due to inconsistent evidence regarding the potential role of vitamin D on lipid profile and sirtuin 1 (SIRT-1), this study was designed to investigate the effect of vitamin D supplementation in combination with weight loss diet on lipid profile and SIRT-1 in obese subjects with vitamin D deficiency.

**Methods:** Forty-four obese subjects with vitamin D deficiency were randomly assigned in a randomized clinical trial to receive either a weight reduction diet supplemented with 50000IU vitamin D3 pearl (n = 22) or placebo (n = 22) once weekly for 12 weeks. Changes in total cholesterol (TC), low-density lipoprotein cholesterol (LDL-C), triglyceride (TG) and low high density lipoprotein cholesterol (HDL-C) and SIRT-1 were the primary outcomes. Secondary outcomes were changes in body mass index (BMI), 25(OH) D and parathyroid hormone (PTH). Physical activity and dietary intakes were also assessed.

**Results:** During the intervention, PTH (mean difference, -33.36; 95% CI: -49.15 to -17.57;P<0.001) and LDL-C (mean difference, -15.91; 95% CI: -21.76 to -10.07; P<0.001) decreased and 25(OH) D (mean difference, 36.44; 95% CI: 29.05 to 43.83; P<0.001) increased significantly in the vitamin D group. BMI (mean differences: -2.40; 95% CI: [-2.92 to-1.88] in vitamin D group and mean differences: -1.90; 95% CI [-6.58 to -3.01] in placebo group, P<0.05 for both groups), TC (mean difference,-21.31; 95% CI: -27.24 to -15.38; P<0.001 in vitamin D group and mean difference, -12.54; 95% CI: -19.02 to -6.06; P<0.001 in placebo group) and TG (mean difference,-21.31; 95% CI: -27.24 to -15.38; P<0.001in vitamin D group and mean difference, -12.54; 95% CI: -19.02 to -6.06; P<0.001 in placebo group) decreased and SIRT-1(mean difference, 3.95; 95% CI: 1.18 to 6.73; P=0.007in vitamin D group and mean difference,1.91; 95% CI: 0.31 to 3.63 in placebo group, P=0.022) increase significantly in both group. At end of the study, 25(OH) D and PTH showed significant differences in between-group analyses(P<0.05). No significant difference was detected for HDL-C in within and between groups.

**Conclusion:** This study gives no support for any beneficial effect of vitamin D supplementation on lipid profile and SIRT-1 in obese subjects with vitamin D deficiency.

## Introduction

Obesity is one of the most important public health problems in the last decade.^1^ The worldwide prevalence of overweight and obesity has been increasing that nearly a third of the world’s population is now suffering from overweight or obese.^2^ Obesity is known to be an expansion of white adipose tissue (WAT) that associated with not only higher plasma triglyceride (TG), total cholesterol (TC), low-density lipoprotein cholesterol (LDL-C), and lower high-density lipoprotein cholesterol (HDL-C)^3^ but also the development of non-communicable diseases risk factors such as hypertension (HTN), dyslipidemia and cardiovascular diseases (CVD).^4,5^ Being a strong risk, hyperlipidemia doubles the risk of CVD development in hyperlipidemic people.^6-8^

Sirtuins (SIRTs) – a class III histone deacetylases, nicotinamide adenine dinucleotide (NAD) dependent enzymes- removes acetyl groups from lysine residues in proteins.^9^ SIRTs have seven categories and SIRT-1 plays an important role in modulating metabolic process including energy homeostasis and adipose tissue metabolism.^10,11^ SIRT-1 inhibits adipogenesis by deacetylation of peroxisome proliferator-activated receptor gamma and stimulating lipolysis, which results in lower levels of adipogenesis.^12^ Moreover, SIRT-1 plays an important role in lipid metabolism and hyperlipidemia by influencing the secretion and action of insulin, which promotes fatty acids storage in WAT and suppresses β-oxidation in the liver and skeletal muscle.^13^ Some studies have shown that SIRT-1 activation leads to deacetylation of liver X receptor proteins, transcription factors that act as cholesterol sensors and regulate whole-body cholesterol, lipid homeostasis and HDL-C production.^14^ New research indicates that SIRT-1 is potential targets for treating CVD.^15^

Vitamin D deficiency is a global public health problem that affects almost 50% of the population worldwide.^16^ A growing body of research indicates that vitamin D is an important factor in the development of CVD.^17^ Recent researches have shown that vitamin D deficiency was closely linked to atherogenic lipid profile that increased risk of adverse CVD events.^18^ High serum 25-hydroxyvitamin D [25(OH) D] levels were related with a suitable serum lipid profile.^19^Although several epidemiologic studies have shown an inverse association between serum vitamin D level and CVD risk and lipid profile, the results of clinical trials are inconsistent.^20^ There are few studies investigating the effect of vitamin D on SIRT-1. The results of an *in vitro* study, vitamin D treatment increased SIRT-1 levels in human umbilical vein endothelial cell.^21^ Chang et al^22^ reported a significant decrease in SIRT-1 activity in obese rats fed with a vitamin D-insufficient diet.

In general, due to inconsistent results about the effects of vitamin D on lipid profile and the shortage of existing data on effect of vitamin D on SIRT-1, the present clinical trial was designed to assess the effects of vitamin D supplementation on serum lipid profile and SIRT1 level in obese subjects with vitamin D deficiency.

## Materials and Methods

### Study design

This double-blind placebo-controlled randomized parallel clinical trial was designed to assess the effect of vitamin D supplementation in combination with weightreduction diet on lipid profile and SIRT-1 among obese subjects with vitamin D deficiency. The obese participants were allocated by a statistician to one of the following groups using computerized random block allocation; “*vitamin D group*” (receiving weight loss diet + a bolus dose of 50000 IU cholecalciferol) or “*placebo group*” (receiving weight loss diet + placebo pearls contained edible paraffin). The participants were asked to take their supplements every Friday right after lunch for 12 weeks. Weight loss diet was designed based on individual characteristics and Basal metabolic rate (BMR) was estimated using Mifflin equations. To estimate total energy expenditure (TEE), after calculating the BMR and also considering individual physical activity level, the BMR was multiplied by 1.5 to 2.1.^23^ Then estimated TEE minus 700 kcal was prescribed for each subject following 12 consecutive weeks.

The goals of weight loss diet program were: daily caloric restriction of 700 kcal, 20-30% fat, 10%-15% protein and 55%-65% carbohydrates. Vitamin D pearls and placebos were made by Zahravi Pharm. Co; Tabriz, Iran. The participants, investigators and the laboratory staff remained blinded until after data collection and statistical analysis. Study follow-up visits were every 2 weeks. Compliance was assessed by counting of the pearls at the end of the study. This study was conducted between October 2016 and March 2017, during which cutaneous vitamin D3 synthesis is minimal.

### Subjects

Participants aged 18-59 years old, with body mass index**(**BMI) ranging from 30 to 40 kg/m^2^, and serum 25(OH) D < 50 nmol/L were recruited by advertisements from Specialized and Sub-specialized Sheykhoraeis clinic of Tabriz University of Medical Sciences, Tabriz. Those who were menopause, pregnant and lactating or professional athlete, having alcohol abuse, taking any medications for lowering lipid, glucose and blood pressure as well as drugs affecting vitamin D metabolism such as cod liver oil, multivitamin- mineral and vitamin D supplements three months before starting the study and during the study period, followed a weight loss diet three months before our study, take weight loss drugs, and metabolic disorder were excluded.

### Sample size calculation

By considering α = 0.05 and power 80%, with an approximate drop-out rate of 10% during the study, the sample size was calculated as 22 per group.^24^

### Measurements

Demographic characteristics including age, sex, marital status, educational level and physical activity status were obtained from each subject at baseline. Height and weightwere measured with light clothes and without shoes using a wall-mounted stadiometer to the nearest 0.1 cm and digital scales to the nearest 0.1 kg using a digital scale (Seca scale, Hamburg, Germany), respectively; BMI was calculated as weight (kg) divided by squared height (m^2^).^25^ A three-day dietary record was used to assess dietary intakes at baseline and end of the study. To assess macro- and micro-nutrient contents of food, Nutritionist IV software (First Databank Inc., Hearst Corp., San Bruno, CA, USA) was applied. Indeed, physical activity levels were evaluated by using the short form of the International Physical Activity Questionnaire (IPAQ) at baseline and after 12 weeks study duration and then categorized as *“Inactive”*, *“Minimally active”*, and *“HEPA (health enhancing physical activity; a high active category”* activity.^26^ The criteria for these three levels are:

1. Low active: No or some activity is reported but not enough to meet Categories 2 or 3.
2. Minimally active: Any one of the following 3 criteria: 3 or more days of vigorous activity of at least 20 minutes per day or 5 or more days of moderate-intensity activity or walking of at least 30 minutes per day or 5 or more days of any combination of walking, moderate or vigorous intensity activities achieving a minimum of at least 600 MET (metabolic equivalent) -min/wk.
3. HEPA active: Any one of the following 2 criteria: Vigorous-intensity activity on at least 3 days and accumulating at least 1500 MET min/wk or more days of any combination of walking, moderate-intensity or vigorous intensity activities achieving a minimum of at least 3000 MET-min/wk.

A standard pre-tested questionnaire was filled by the participants to record how long they are exposed to the sun. The participants were asked to write the how many minutes/hour they spent in daylight during the last 7 days.^27^

After a 12 hour overnight fast, blood samples (10 mL) were collected from the antecubital vein into the vacutainer tubes. After centrifugation for 20 minutes (3000 g), the serum samples were frozen consecutively and kept at -80°C until the day of analysis.

Serum levels of 25(OH) D, parathyroid hormone (PTH) and SIRT-1 were assessed using high sensitivity enzyme linked immune-sorbent assay (ELISA) kit (crystal day, shanghai); intra- and inter-assay coefficients of variation were 8% and 10%, respectively. Serum TG, TC and HDL-C concentrations were determined enzymatically (Parsazmon, Tehran) and then Friedewald equation was used for calculation of LDL-C among those with serum TG level <400 mg/dL as follows^28^:

(LDL-C = total cholesterol – HDL-C − [TG/5])

Calcium and phosphorus were measured by colorimetric enzymatic (Diagnostic Chemicals Limited, San Diego, CA, USA) (normal range 2.15-2.57 mmol/L. the assay sensitivity 0.05 mmol/L and intra- and inter-assay coefficient of variation were 2.4% and 3%, respectively).

### Statistical analysis

All statistical analyses were performed by IBM-SPSS-Statistics version 23.0 (IBM, Armonk, New York, USA). Numerical variables data are presented as mean ± standard deviation (SD) and categorical variables were presented as frequency (percentage). Kolmogorov-Smirnov test was used for examination normality of data. All data had normal distribution, therefore parametric tests were performed. Independent-samples *t* test and chi-square tests were applied for comparison of baseline data between the two groups (for continuous and categorical variables, respectively) and paired samples *t* test was used for assessment of intragroup changes before and after the study. Between-groups differences were compared at the end of the study using analysis of covariance (ANCOVA) adjusted for baseline values as well as covariates such as serum calcium concentrations, changes on weight, dietary intake of vitamin D and sun exposure. *P* value < 0.05 was considered as statistically significant.

## Results

### Subject characteristics

**Figure 1:**
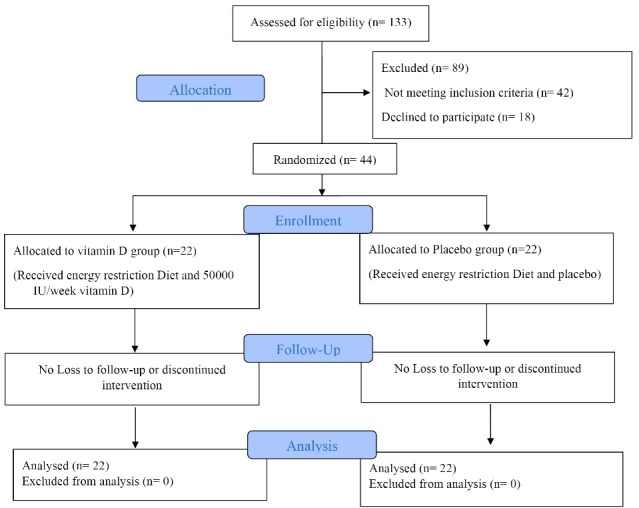
Study flow diagram.

Figure 1 shows the number of individuals assessed for eligibility (n = 133), randomized (n = 44) and included in the analysis (n = 44). All the participants completed the study. The compliance to the 12-week intervention for both groups was 97%. No adverse effect was reported after supplementation. The characteristics of participants are shown in Table 1. Baseline characteristics were similar in studied groups (*P*> 0.05).

### Anthropometric measurements

**Figure 2:**
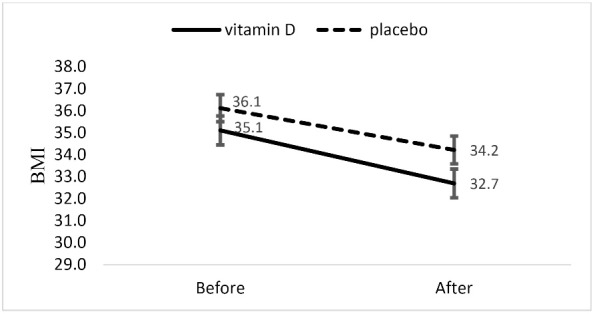
BMI before and after intervention in vitamin D and placebo groups.

As shown in Figure 2, BMI decreased significantly in both groups (mean differences: -2.40; 95% CI [-2.92 to-1.88] in vitamin D group and mean differences: -1.90; 95% CI [-6.58 to -3.01] in placebo group, *P* < 0.05 for both groups).

No significant difference in changes in this variable was observed between two groups after adjusting for baseline values and confounders.

### Dietary intake

As shown in Table 2, energy intake decreased significantly in both groups The ratio of protein intake rose from 9.18% of total calorie at week 0 to 11.31% at week 12 in vitamin D group (*P*< 0.05), also protein portion of the total calories increased from 6.83% to 12.58% in the placebo group (*P* < 0.05). Change in total fat content of diet was 18.58% to 27.39 in vitamin D group and 16.08% to 27.02% in placebo group (*P* < 0.05). Carbohydrates constitute 72.23% of total calories in vitamin D group and 77.09% in placebo group initially, that this amount was reduced to 61.3% and 64.4%, respectively (*P* < 0.05). Energy, macronutrients and vitamin D intake did not differ between the two groups during the 12-week energy-reduction diet.

### Vitamin D-related serum biomarkers

The mean serum 25(OH) D level showed significant increase in vitamin D group (mean difference, 36.44; 95% CI: 29.05 to 43.83; *P* < 0.001) while the placebo group showed no significant changes for vitamin D status (mean difference, 2.9; 95% CI: -0.41 to 6.30; *P*˃ 0.05). 25(OH) D level between the two groups showed significant difference (*P* < 0.001). Mean serum PTH decreased significantly in vitamin D group (mean difference, -33.36; 95% CI: -49.15 to -17.57; *P* < 0.001) with no changes in placebo group (mean difference, -10.28; 95% CI: -28.28 to 7.71; *P*˃ 0.05). In between group analysis, a significant difference was observed for PTH concentration at the end of the study (*P*= 0.007; Table 3).

### Lipid profile and SIRT-1

After 12 weeks, TG showed significant decreases in both groups compared to baseline (mean differences: -20.77; 95% CI [-32.94 to -8.59]; *P*=0.002 in vitamin D group and mean differences: -16.04; 95% CI [-25.05 to -7.04]; *P*< 0.001 in placebo group). There was a reduction in the mean serum TC level in both group (mean difference, -21.31; 95% CI: -27.24 to -15.38; *P* < 0.001) in vitamin D group and (mean difference, -12.54; 95% CI: -19.02 to -6.06; *P* < 0.001) in placebo group. Serum LDL-C decreased (mean difference, -15.91; 95% CI: -21.76 to -10.07;*P* < 0.001) in vitamin D group; no significant changes were found in this parameter in the placebo group (mean difference, -6.25; 95% CI: -13.33 to 0.81;*P*> 0.05). After 12 weeks intervention, HDL-C was not significantly different compared to baseline in both group (*P*> 0.05). Comparison of lipid profile between the two groups, revealed no significant difference at end of the study (*P*> 0.05; Table 3).

As shown in Table 3,SIRT-1 increased significantly in both group (mean difference, 3.95; 95% CI: 1.18 to 6.73; *P*= 0.007) and placebo group (mean difference, 1.91; 95% CI: 0.31 to 3.63 in placebo group; *P*= 0.022); however, no significant differences were observed between the groups (*P*> 0.05).

## Discussion

The results of the present RCT revealed no significant effect of 12 weeks vitamin D supplementation in combination of energy restriction on serum lipids profile and SIRT-1 in obese subjects with vitamin D deficiency.

Our results indicated that vitamin D supplementation and weight loss diet for 12 weeks in obese subjects significantly decreased TC and TG in both group and LDL-C in vitamin D group, but no significant differences were observed for lipid profile between the groups.

In current study, the percent change of weight loss in the vitamin D and placebo groups was -6.89% and -4.47% respectively. A randomized clinical trial revealed that even a moderate 5% weight loss has considerable health benefits, including decreased plasma TG concentration, lipid synthesis and lipid cholesterol flux.^29^ It seems reduction in TC and TG in both groups is related to weight loss.

Although observational studies showed that high 25(OH) D levels were associated with a favorable serum lipid profile,^30^ interventional studies provided divergent results.The results of this research are in agreement with the recently published research byKubiak et al^31^ in 2019 which found no effect of high-dose vitamin D (100 000 IU loading dose, followed by 20 000 IU/wk) on serum lipid profile in vitamin D-insufficient subjects. Similarly, Wamberg et al^32^ reported no effects of daily 7000 IU vitamin D for 26 weeks on lipid profile in obese adults with low vitamin D levels. However,Jorde et al^33^ study in 438 overweight or obese subjects randomized to vitamin D 40 000 IU/wk, vitamin D 20 000 IU/wk, or placebo revealed no important differences between the three groups regarding change in measures of serum lipids and other cardiovascular risk factors. A recent meta-analysis in 2019 concluded no statistically significant effect of vitamin D supplementation on TC, TG and LDL-C among participants with CVD.^34^ In contrast, an RCT performed by Mohamad et al^35^ reported that 4500 IU/day vitamin D supplementation decreased TG levels in diabetic females. Also,findings of Ramiro-Lozano and Calvo-Romero^36^ presented that oral weekly supplementation of vitamin D for eight weeks (16 000 IU) showed reduction in TC but not LDL-C and TG in participants with type 2 diabetes. The possible reasons for the results inconsistency could be due to the difference in the characteristics of the studied population, dose and the duration of vitamin D supplementation as well as baseline serum levels of vitamin D and lipid profile.

In the current trial, serum levels of SIRT-1 increased significantly in both groups but differences between groups were not significant. A variety of studies have demonstrated that weight loss associated with up- regulation of SIRT-1. These results approve animal studies that found elevated expression of SIRT-1 genes in the adipose tissue of energy-deprived mice.^37^

Although the beneficial effects of vitamin D on SIRT-1 have been shown in *in vitro* and animal studies, there are limited clinical trials. An *in vitro* experiment indicated that vitamin D up-regulated SIRT-1 and reverted the SIRT-1 down-regulation induced by H2O2 in human endothelial cells.^21^ Moreover, vitamin D increased the expression and activity of SIRT-1 in 3T3-L1 adipocytes.^22^ In line with these findings, Chang and Kim^38^ study described that vitamin D deficiency significantly decreased SIRT-1 in obese rats fed with a vitamin D-insufficient diet. A possible underlying mechanism of the relationship between vitamin D and SIRT-1 could be the capacity of vitamin D to increase adenosine monophosphate-activated protein kinase which enhances SIRT-1 by increasing NAD/NADH ratio and decreases adipose tissuemacrophage infiltration and inflammation.^39^

Although the present study has some limitations such as relatively small sample size and duration of the treatment, the strengths of this study are methodology (i.e. a double-blind, randomized, placebo-controlled design) which allows for causative conclusions in both genders. Indeed, all subjects received a standard diet (20%-30% fat, 10%-15% protein and 55%-65% carbohydrates), and therefore, diet composition was not considered as a confounding factor.

## Conclusion

In conclusion, the results of this RCT suggest that weekly supplementation with 50 000 IU vitamin D in combination with a weight loss diet have no effect on serum lipid profile and SIRT-1 in obese subjects with vitamin D deficiency. Further well-designed RCT with relatively larger sample size and longer follow-up period are needed to understand the effect of vitamin D supplementation on lipid profile and SIRT-1 in obese patients with vitamin D deficiency.

## Ethical approval

A written informed consent was signed by all participants. The trial was conducted according to the principles of the Declaration of Helsinki and received ethical approval from Tabriz University of Medical Science (reference number: TBZMED.REC.1395.761). The present trial was registered in Iranian Registry of Clinical Trial website (identifier: IRCT201608223320N13; http://www.irct.ir).

## Competing interests

The authors declare that they have no competing interests.

## Funding

This work was supported by the Research Vice Chancellor, Tabriz University of Medical Sciences.

## Authors’ contributions

Author contributions were as follows: Study concept and design (SA, MEM, SRAH); Acquisition of data (SA, LLD); Analysis and interpretation of data (SA, MEM Drafting of the manuscript (SA, MEM, SRAH and LLD) and Critical revision of the manuscript for important intellectual content (MEM, SRAH); All authors read and approved the final manuscript.

## Acknowledgments

The authors would like to thank the all patients who participated as samples of this study. We, also appreciate Zahravi (Zahravi Pharm. Co; Tabriz, Iran) for providing vitamin D and placebo pearls. This article is provided from Ph.D. thesis of Soodabeh Aliashrafi with the registered number of (D/52) at Tabriz University of Medical Sciences.

**Table 1:** Baseline characteristics of study groups

**Variables**	**Vitamin D** **(n =22)**	**Placebo** **(n =22)**	***P***
Age, (y)	35.18± 7.00	34.90 ± 10.37	0.919^a^
Male, No. (%)	9 (22.5)	10 (22.7)	0.761^b^
Weight (kg)	99.60 ± 13.95	99.65 ± 14.15	0.99^a^
Height (cm)	168.31 ± 9.45	166.4 ± 7.25	0.457^a^
25(OH) D (nmol/L)	28.70 ± 13.83	25.02 ± 12.72	0.363^a^
Calcium (mg/dL)	8.62 ± 0.40	8.91 ± 0.48	0.034^a^
Sun exposure, No. (%)			0.258^b^
None	14 (63.6)	11 (50)	
10 min–1 h	8 (36.4)	7 (31.8)	
1-2 h	0 (0)	3 (13.8)	
>2 h	0 (0)	1 (4.5)	
Physical activity, No. (%)			0.345^b^
Low active	21 (95.5)	18 (81.8)	
Minimally active	1 (4.5)	4 (18.2)	
HEPA	0 (0)	0 (0)	

HEPA, health enhancing physical activity. Data presented as mean ± standard deviation for quantitative variable and No. (%) for categorical variables. ^a^ *P* value for independent sample *t* test. ^b^ *P* value for chi-square test.

**Table 2:** Dietary intakes of the study group at baseline and after 12 weeks intervention

Variable*	**Baseline values**		**After 12 weeks intervention**		***P*** **value** ^a^	***P*** **value** ^a^	***P*** **value** ^b^
	**Vitamin D group**	**Placebo group**	**Vitamin D group**	**Placebo group**	**(Difference within vitamin D group)**	**(Difference within placebo group)**	**(Difference between groups)**
Energy (kcal/days)*	2452.59 ± 624.94	2356.86 ± 537.12	1628.59 ± 429.38	1567.40 ± 386.29	< 0.0001	< 0.0001	0.862
Protein (% of energy)	9.18	6.83	11.31	12.58	< 0.0001	< 0.0001	0.854
Fat (% of energy)	18.58	16.08	27.39	23.02	< 0.0001	< 0.0001	0.528
Carbohydrate (% of energy)	72.23	77.09	61.3	64.4	< 0.0001	< 0.0001	0.249
Vitamin D (µg/d)	0.76 ± 0.66	0.46 ± 0.43	0.64 ±.056	0.45 ± 0.38	0.059	0.907	0.669
Calcium (mg/d)	717.33 ± 202.88	714.99 ±273.38	698.93 ± 180.01	615.12 ± 48.57	0.600	0.117	0.34
Phosphorous (mg/d)	744.48 ± 174.75	801.36 ± 189.28	707.78 ± 209.56	684.43 ± 171.28	0.384	0.155	0.270

* Mean ± SD. ^a^ *P* value for paired *t* test. ^b^ *P* value for ANCOVA; adjusted for baseline values.

**Table 3:** Vitamin D related biomarkers, serum lipid profile and Sirtuin 1 in study groups at baseline and end of 12 weeks intervention

Variable*	**Baseline values**		**After 12 weeks intervention**		***P*** **value** ^a^	***P*** **value** ^a^	***P*** **value** ^b^
	**Vitamin D group** **(n=22)**	**Placebo group** **(n=22)**	**Vitamin D group (n=22)**	**Placebo group (n=22)**	**(Difference within** **vitamin D group)**	**(Difference within** **placebo group)**	**(Difference between groups)**
25(OH) D (nmol/L)	28.70 ± 13.83	25.02 ± 12.72	65.14 ± 17.88	27.96 ± 14.37	<0.001	0.083	<0.001
PTH (pg/mL)	92.26 ± 36.46	94.30 ± 37.50	58.90± 0.01	84.02 ± 24.21	<0.001	0.248	0.007
Calcium (mg/dL)	8.62 ± 0.40	8.91 ± 0.48	8.55 ± 0.42	8.83 ±0.52	0.442	0.453	0.357
Phosphorus (mmol/L)	3.70 ± 0.46	3.51 ± 0.36	3.80 ±0.62	3.33 ± 0.65	0.446	0.081	0.105
TG (mg/dL)	135.04 ± 41.15	125.22 ± 41.36	119.00 ± 38.66	104.45 ± 33.61	0.002	<0.001	0.803
TC (mg/dL)	181.81 ± 12.92	179.13 ± 16.28	160.5±13.91	166.59 ± 20.75	<0.001	<0.001	0. 311
HDL-C (mg/dL)	48.8±16.98	42.66±4.52	48.27±11.67	42.43±4.07	0.85	0.767	0.162
LDL-C (mg/dL)	108.17 ± 14.83	98.46 ± 20.19	92.25 ± 11.71	92.20 ± 20.71	<0.001	0.08	0.679
SIRT-1 (ng/mL)	33.89 ± 22.25	37.85 ± 23.93	32.47 ± 17.85	34.45 ± 17.04	0.007	0.022	0.705

PTH, Parathyroid hormone; TC, Total cholesterol; LDL-C, low-density lipoprotein cholesterol, TG, triglyceride; HDL-C, low high-density lipoprotein cholesterol, SIRT-1: Sirtuin 1. * All values are mean ± SD. ^a^ *P* value for Paired t test. ^b^ *P* value for ANCOVA; adjusted for baseline values, serum calcium concentrations, changes on weight, dietary intake of vitamin D and sun exposure.
